# TLP-mediated global transcriptional repression after double-strand DNA breaks slows down DNA repair and induces apoptosis

**DOI:** 10.1038/s41598-019-41057-9

**Published:** 2019-03-19

**Authors:** Hidefumi Suzuki, Mayumi Okamoto-Katsuyama, Tetsufumi Suwa, Ryo Maeda, Taka-aki Tamura, Yuki Yamaguchi

**Affiliations:** 1 School of Life Science and Technology, Tokyo Institute of Technology, 4259 Nagatsuta, Yokohama, 226-8501 Japan; 2 Graduate School of Science, Chiba University, 1-33 Yayoicho, Chiba, 263-8522 Japan

## Abstract

Transcription and DNA damage repair act in a coordinated manner. Recent studies have shown that double-strand DNA breaks (DSBs) are repaired in a transcription-coupled manner. Active transcription results in a faster recruitment of DSB repair factors and expedites DNA repair. On the other hand, transcription is repressed by DNA damage through multiple mechanisms. We previously reported that TLP, a TATA box-binding protein (TBP) family member that functions as a transcriptional regulator, is also involved in DNA damage-induced apoptosis. However, the mechanism by which TLP affects DNA damage response was largely unknown. Here we show that TLP-mediated global transcriptional repression after DSBs is crucial for apoptosis induction by DNA-damaging agents such as etoposide and doxorubicin. Compared to control cells, TLP-knockdown cells were resistant to etoposide-induced apoptosis and exhibited an elevated level of global transcription after etoposide exposure. DSBs were efficiently removed in transcriptionally hyperactive TLP-knockdown cells. However, forced transcriptional shutdown using transcriptional inhibitors α-amanitin and 5,6-dichloro-1-ß-D-ribofuranosylbenzimidazole (DRB) slowed down DSB repair and resensitized TLP-knockdown cells to etoposide. Taken together, these results indicate that TLP is a critical determinant as to how cells respond to DSBs and triggers apoptosis to cells that have sustained DNA damage.

## Introduction

It has been reported that transcription and DNA damage repair act in a coordinated manner. Active transcription accelerates DNA damage repair through multiple mechanisms. In transcription-coupled nucleotide excision repair, bulky base adducts, such as pyrimidine dimers, induced by UV light or environmental mutagens are removed preferentially in actively transcribed genes^1^. Moreover, recent studies revealed that DSBs are also removed more efficiently in actively transcribed genes^2–5^. Transcriptionally engaged RNA polymerase II (RNAPII) recruits factors involved in homologous recombination (HR) repair to damaged sites^2^. Furthermore, it is demonstrated that nascent RNA is used as a template for HR repair^4,5^. Thus, transcription plays an important role in the repair of damaged DNA.

On the other hand, DNA damage globally represses transcriptional activity by multiple pathways^6–9^. Shanbhag *et al*. showed that ATM-dependent ubiquitination of histone H2A-K119 results in the prevention of RNAPII elongation at DSB sites^6^. Kakarougkas *et al*. showed that the chromatin remodeling complex PBAF is important for DSB-induced transcriptional repression^8^. However, the molecular mechanism underlying DNA damage-induced transcriptional repression is not fully understood. Regardless, it is speculated that global transcriptional repression facilitates DNA repair by eliminating RNAPII, which could interfere with DNA repair, from damaged DNA^6,8,9^.

The anti-cancer drug etoposide is a DNA topoisomerase II (Topo II) inhibitor that induces DSBs^10,11^. Topo II catalyzes a two-step reaction involving DNA scission. A covalent link between Topo II and cleaved DNA is formed in the first step, and etoposide accumulates this intermediate complex by inhibiting the second step of the catalytic reaction. Since Topo II functions mainly during DNA replication, Topo II inhibition by etoposide is thought to affect cells principally in S phase^12^. Etoposide-induced DSBs activate ATM and ATR, which then phosphorylate their target proteins including CHKs and p53 to induce cell cycle arrest. G1- or G2-arrested cells attempt to remove DSBs by activating DNA repair pathways, and failure in DSB repair results in apoptosis^11,13,14^. DSBs can be repaired by two alternative pathways, namely non-homologous end joining (NHEJ) and HR^15–17^. NHEJ is an error-prone repair mechanism driven by proteins including Ku70/80, DNA-PK, and XRCC4. On the other hand, HR is an error-free repair mechanism driven by proteins including CtIP, Rad51, Rad52, and BRCA2. NHEJ is active throughout cell cycle and is thought to be a dominant repair pathway. Meanwhile, HR repair is active only in S and G2 phase and critical as a backup repair pathway^15,16^. Since etoposide mainly induces DSBs during DNA replication in S phase, HR repair can be critical for repairing etoposide-induced DSBs.

TBP-Like Protein (TLP), also known as TBP-Related Factor 2 (TRF2), is a member of the TBP family that includes TRF1 and TRF3 (TBP2)^18–21^. TBP family proteins exhibit similarity in structure and function to TBP, and TLP has the lowest sequence similarity to TBP among them. Whereas TRF3 is functionally similar to TBP with respect to TATA box-binding activity^20,21^, TLP lacks sequence-specific DNA-binding activity but has the highest affinity to TFIIA^22,23^. It was shown that TLP inhibits TBP function by sequestering TFIIA^24^. Recent studies on the *Drosophila* homolog of TLP have shown that it is also recruited to some TATA-less genes by sequence-specific DNA-binding proteins and activates transcription^25–27^. Moreover, there are several lines of evidence suggesting that TLP is involved in DNA damage response. Subcellular localization of TLP is altered by various DNA-damaging agents, and the expression level of TLP is upregulated by DSBs^28,29^. TLP is important for cellular response to UV and etoposide, as its knockdown attenuates their cytotoxic effects^30,31^. It was shown that TLP activates *p63* expression and accelerates apoptosis induction in etoposide-treated cells^30^; however, the physiological significance of TLP-mediated transcriptional repression in DNA damage response is not well understood. In this study, we investigated the role of TLP-mediated transcriptional repression in etoposide-induced DNA damage response. Here we show that TLP-mediated transcriptional repression is involved in etoposide-induced apoptosis through modulating DNA damage repair activity. Compared to control cells, TLP-knockdown cells exhibited resistance to etoposide-induced apoptosis and global transcriptional de-repression after etoposide exposure. Etoposide-induced DSBs were efficiently repaired in transcriptionally hyperactive TLP-knockdown cells. Moreover, forced transcriptional shutdown using transcriptional inhibitors α-amanitin and DRB delayed DSB repair and resensitized TLP-knockdown cells to etoposide. Taken together, these results indicate that TLP-mediated transcriptional repression plays an important role to determine sensitivity to etoposide-induced DNA damage.

## Results

### TLP is required for etoposide-induced apoptosis induction

**Figure 1:**
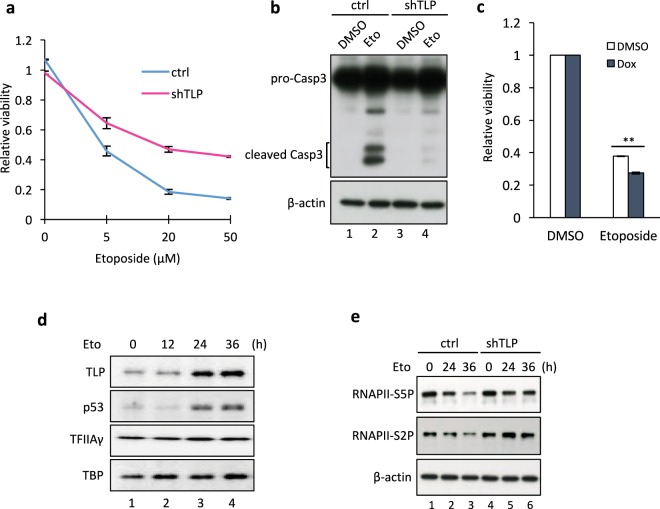
TLP is required for etoposide-induced apoptosis induction. (**a**) Cell viability of etoposide-treated cells. Control (ctrl) and TLP-knockdown (shTLP) HeLa cells were treated with indicated concentrations of etoposide for 36 h, and cell viability was determined by SF assay. Data were normalized to the level of nontreated cells and represent the average and S.D. of three independent experiments. (**b**) Caspase-3 cleavage after etoposide treatment. The conversion of Procaspase-3 (pro-Casp3) to Caspase-3 (Casp3) was monitored by Western blotting 24 h after etoposide treatment. (**c**) The effect of TLP overexpression on etoposide sensitivity. TLP overexpression was induced by adding 100 ng/ml doxycycline (Dox) to shTLP-TetOn cells. Control and TLP-overexpressing cells were treated with etoposide for 36 h, and cell viability was determined by SF assay. Data were normalized to the level of DMSO-treated cells and represent the average and S.D. of three independent experiments. **p < 0.01. (**d**,**e**) HeLa cells were treated with 50 μM etoposide for indicated periods and subjected to Western blot analysis.

Exposure of cells to genotoxic agents such as ionizing radiation and the Topo II inhibitor etoposide results in cell growth arrest and apoptosis. We previously reported that siRNA-mediated TLP knockdown confers resistance to etoposide^30–33^. To confirm this, we examined etoposide sensitivity of cells in which TLP expression was stably knocked down. As expected, stable TLP knockdown conferred etoposide resistance. After continuous etoposide treatment, TLP-knockdown cells exhibited a significantly higher viability than control cells (Fig. 1a). Etoposide-induced cleavage of Caspase 3, a marker of apoptosis induction, was markedly suppressed in TLP-knockdown cells (Fig. 1b).

Cell cycle analysis showed that etoposide treatment increased sub-G1, S, and G2/M population in control cells (Supplementary Fig. S1), in agreement with previous findings^34,35^. Moreover, TLP knockdown partially suppressed the increase in sub-G1 and S population by etoposide treatment for 24 h, suggesting that TLP is involved in etoposide-induced apoptosis and S-phase arrest. On the contrary, G2/M population was slightly increased rather than decreased by TLP knockdown, suggesting that TLP may contribute to mitotic progression. Incidentally, TLP knockdown cells have a slightly slower growth rate than control cells, with doubling times of approximately 21.6 hours and 18.5 hours, respectively (Supplementary Fig. S2a), which may be due to a slower mitotic progression of TLP knockdown cells.

We also examined the effect of a brief treatment with etoposide and found that etoposide treatment for as short as 30 min was sufficient to induce significant growth inhibition (Supplementary Fig. S2b). Moreover, TLP knockdown attenuated etoposide-induced growth inhibition in all the cases we examined (Supplementary Fig. S2b), further substantiating its critical role in etoposide-induced apoptosis. These results prompted us to examine whether TLP-knockdown cells are also resistant to various other stresses, such as γ irradiation, doxorubicin (another Topo II inhibitor), the alkylating agent methyl methane-sulfonate (MMS), and the proteasome inhibitor MG132. As a result, TLP-knockdown cells were found to be resistant to γ irradiation and doxorubicin, but not to MMS and MG132 (Supplementary Fig. S2c,d), suggesting that TLP knockdown confers resistance to genotoxic stresses that induce DSBs. To validate the above findings, TLP expression was induced using the Tet-On system. Re-expression of TLP in knockdown cells increased sensitivity to etoposide and doxorubicin (Fig. 1c and Supplementary Fig. S1e), suggesting that TLP is a critical determinant as to how cells respond to these DNA-damaging agents.

Consistent with our previous study^30,32,33^, the TLP protein level was dramatically increased by etoposide treatment (Fig. 1d). Meanwhile, DNA DSBs caused by Topo II inhibition are known to repress transcription^36^. To investigate a possible role of TLP in DNA damage-induced transcriptional repression, we examined the phosphorylation status of the RNAPII C-terminal domain (CTD). Ser-5 and Ser-2 phosphorylation of the RNAPII CTD is a hallmark of active transcription initiation and elongation, respectively. In control cells, etoposide treatment caused a decrease in the phosphorylation levels of both Ser-2 and Ser-5 (Fig. 1e). In TLP-knockdown cells, however, etoposide had only a modest effect on their phosphorylation levels, suggesting that TLP contributes to etoposide-induced transcriptional repression. TLP expression is induced by 24 h after etoposide addition (Fig. 1d), implying that TLP-dependent transcriptional repression is stronger in cells that have sustained DNA damage.

### TLP is required for etoposide-induced transcriptional repression

**Figure 2:**
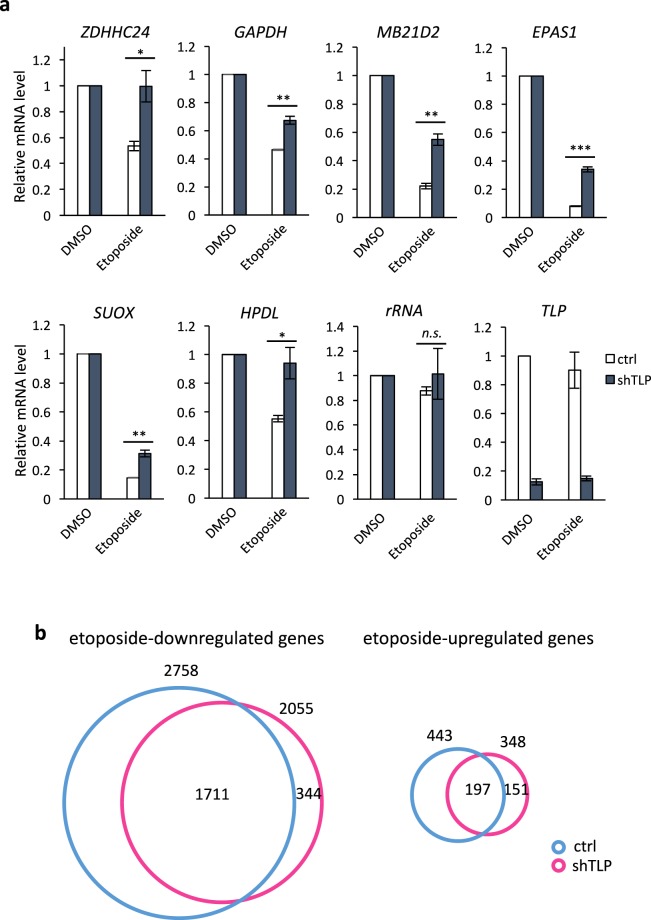
TLP is critical for etoposide-induced changes in gene expression. (**a**) qRT-PCR analysis of control (ctrl) and TLP-knockdown (shTLP) HeLa cells that were treated with DMSO or 50 µM etoposide for 24 h. Data were normalized to the level of DMSO-treated control conditions and represent the average and S.D. of three independent experiments. *p < 0.05; **p < 0.01; ***p < 0.001; n.s, non-significant. (**b**) Control and TLP-knockdown cells were treated with DMSO or 50 μM etoposide for 24 h and subjected to mRNA-seq analysis. Experiments were performed in duplicate, and the expression levels (in fragments per kilobase per million reads) in DMSO- and etoposide-treated cells were compared. Genes with q < 0.05 and log_2_|fold change| ≥ 1.0 were regarded as etoposide-downregulated and -upregulated genes.

To understand the precise role of TLP in etoposide-induced transcriptional repression, we next investigated etoposide-induced transcriptional changes in control and TLP-knockdown cells. Etoposide treatment strongly repressed expression of a subset of genes at the RNA level (Fig. 2a). On the other hand, TLP knockdown significantly attenuated etoposide-induced transcriptional repression of these genes (Fig. 2a). Consistent results were obtained by mRNA-seq analysis, which showed that 2,758 genes and 2,055 genes were significantly downregulated by etoposide in control cells and in TLP-knockdown cells, respectively (Fig. 2b, left panel). Etoposide-downregulated genes were largely overlapped between control and TLP-knockdown conditions, but the number of affected genes was substantially smaller in the latter case, suggesting that etoposide-induced transcriptional repression was impaired by TLP knockdown. Similarly, 443 genes and 348 genes were significantly upregulated by etoposide in control cells and in TLP-knockdown cells, respectively (Fig. 2b, right panel), suggesting that etoposide-induced transcriptional activation was also attenuated by TLP knockdown. GO analysis showed that genes involved in nucleosome assembly and cell-cell adhesion were particularly enriched in etoposide-affected genes; although there was no big difference in the GO terms identified between control and TLP-knockdown conditions (Supplementary Fig. S3a). Taken together, these results suggest that TLP influences global gene expression after etoposide exposure.

Since TATA-containing promoters are thought to be a major target of TLP-mediated transcriptional repression, we investigated the proportion of TATA-containing genes in etoposide-affected genes. Enrichment analysis for transcription factor-binding sites using DAVID showed that 60.2% of top 1,000 etoposide-downregulated genes contain TATA, while only 37.1% of top 1,000 etoposide-upregulated genes contain TATA (Supplementary Fig. S3b). These findings suggest that TATA-containing genes are more prone to downregulation by etoposide, and that TLP is involved in etoposide-induced downregulation of TATA-containing genes.

**Figure 3:**
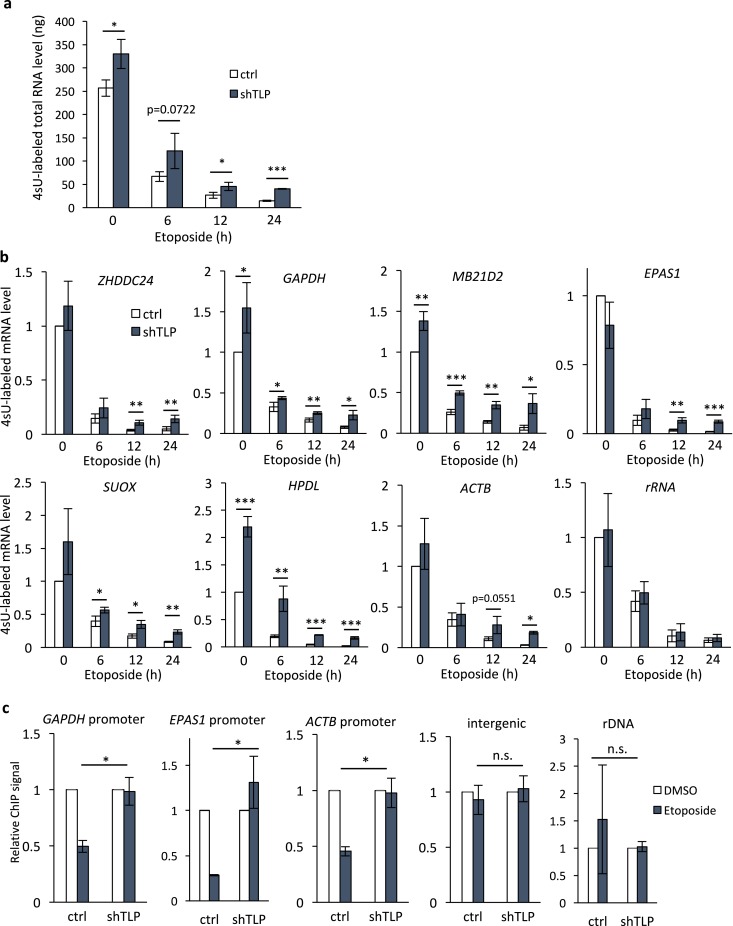
TLP is required for etoposide-induced transcriptional repression. (**a,b**) The rate of ongoing transcription was analyzed by metabolic labeling of newly transcribed RNA using 4sU. 4sU-RNA labeling was performed using control (ctrl) and TLP-knockdown (shTLP) HeLa cells that were treated with 50 μM etoposide for indicated times, and the total quantity of 4sU-labeled RNA was measured with a Quantus fluorometer (**a**). qRT-PCR was performed using 4sU-labeled RNA as a template (**b**). Data were normalized to the level of non-treated cells (0 h) and represent the average and S.D. of three independent experiments. *p < 0.05; **p < 0.01; n.s, non-significant. (**c**) Control and TLP-knockdown HeLa cells were treated with DMSO or 50 μM etoposide for 24 h, and ChIP was performed using anti-RNAPII CTD. Data were normalized to the level of DMSO-treated conditions and represent the average and S.D. of three independent experiments. *p < 0.05, n.s., non-significant.

In principle, RNA expression levels are determined by the equilibrium between RNA synthesis and degradation. To determine whether the etoposide-induced transcriptomic changes were indeed caused by changes in the rate of mRNA synthesis, we investigated the effect of TLP knockdown on ongoing transcription by labeling nascent transcripts using 4-sU. In control cells, the total quantity of RNA newly synthesized in 45 min was decreased to ~4% by 24 h of etoposide treatment (Fig. 3a). When TLP was knocked down, the quantity of newly synthesized RNA was increased 1.28-fold under DMSO-treated conditions and up to 2.85-fold under etoposide-treated conditions. Concordantly, the quantities of newly synthesized mRNAs for multiple genes were significantly increased by TLP knockdown before and after etoposide treatment while rRNA synthesis was unaffected (Fig. 3b), suggesting that the level of ongoing transcription is generally higher in TLP-knockdown cells.

We previously demonstrated that TLP inhibits the initiation step of transcription^24^. To examine whether the etoposide-TLP pathway involves the inhibition of transcription initiation, we indirectly measured promoter activity by ChIP analysis of promoter-bound RNAPII. RNAPII occupancy at the promoter region of *GAPDH*, *EPAS1*, and *ACTB* was significantly reduced by etoposide treatment in control cells, but not in TLP-knockdown cells (Fig. 3c), suggesting that TLP inhibits the recruitment of RNAPII in etoposide-treated cells. Taken together, our observations suggest that TLP mediates etoposide-induced transcriptional shutdown by inhibiting preinitiation complex assembly.

### TLP-mediated transcriptional shutdown after etoposide exposure is crucial for efficient apoptosis induction

**Figure 4:**
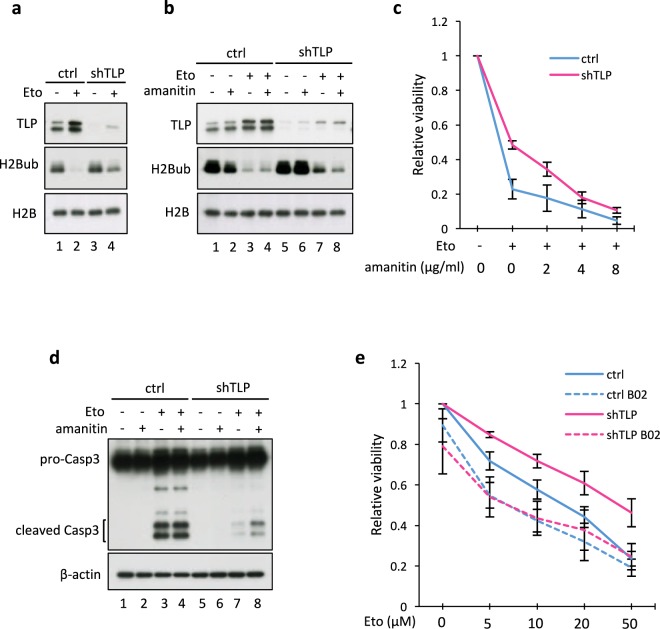
TLP-mediated transcriptional shutdown after etoposide exposure is crucial for efficient apoptosis induction. (**a,b**) Control (ctrl) and TLP-knockdown (shTLP) HeLa cells were treated with 50 μM etoposide (Eto) for 24 h or left untreated (NT), and the total H2Bub level was monitored by Western blotting. Where indicated, α-amanitin was added to the final concentration of 2 µg/ml 12 h before analysis. (**c**) Control and TLP-knockdown HeLa cells were treated with 50 μM etoposide and indicated concentrations of α-amanitin. Cell viability was determined by SF assay and normalized to the cell number under nontreated conditions. The y-axis represents the average and S.D. of three independent experiments. (**d**) Control and TLP-knockdown HeLa cells were treated as in (**b**), and the conversion of Procaspase-3 (pro-Casp3) to Caspase-3 (Casp3) was monitored by Western blotting. (**e**) Etoposide sensitivity of B02-treated HeLa cells. Control and TLP-knockdown HeLa cells were treated with indicated concentrations of etoposide with or without 10 μM B02 for 36 h, and cell viability was determined by SF assay. The y-axis represents the average and S.D. of three independent experiments.

To investigate the possible impact of TLP-mediated transcriptional shutdown on the chromatin structure of etoposide-treated cells, we analyzed the level of H2B mono-ubiquitination (H2Bub). H2Bub is a hallmark of relaxed and transcriptionally active states^37–40^. In control cells, etoposide treatment almost eliminated H2Bub. In TLP-knockdown cells, however, etoposide treatment had only a modest effect on the H2Bub level (Fig. 4a). These results suggest that TLP-knockdown cells have relaxed chromatin structure even after etoposide exposure.

H2Bub is also known as a hallmark of active DNA repair, as H2Bub-containing chromatin is more accessible to DNA repair factors^39,40^. Moreover, active transcription is sometimes critical for efficient DNA damage repair^2–4^. Based on these reports, we hypothesized that an elevated level of transcription in TLP-knockdown cells leads to more efficient DNA repair and thereby results in resistance to etoposide. To test this idea, we examined the effect of the transcriptional inhibitor α-amanitin^41^. Under the conditions employed, α-amanitin alone had little effect on cell viability (Fig. 4d and Supplementary Fig. S4a). Coadministration of α-amanitin with etoposide, however, diminished the level of residual H2Bub in TLP-knockdown cells (Fig. 4b), implying that transcriptional de-repression by TLP knockdown was reversed by α-amanitin. Concordantly, α-amanitin treatment resensitized TLP-knockdown cells to etoposide, whereas increasing concentrations of α-amanitin had a less pronounced effect on the viability of etoposide-treated control cells (Fig. 4c). Moreover, coadministration of α-amanitin with etoposide accelerated Caspase 3 cleavage in TLP-knockdown cells, but not in control cells (Fig. 4d), suggesting that transcriptional de-repression in TLP-knockdown cells is critical for etoposide resistance and for the prevention of etoposide-induced apoptosis. Lack of effect of α-amanitin in control cells is probably because transcription is efficiently repressed by etoposide treatment in control cells.

Next, we examined whether the etoposide resistance of TLP-knockdown cells is due to increased efficiency of DNA repair. To this end, we used B02, the RAD51 inhibitor that blocks DSB-induced HR^42^. B02 alone is toxic to cells and induced apoptosis in a concentration-dependent manner, with no apparent difference in sensitivity between control and TLP-knockdown cells (Supplementary Fig. S4b). Therefore, 10 µM B02, which had little effect on cell viability alone, was used in combination with various concentrations of etoposide. While B02 had only a weak, if any, effect on etoposide sensitivity of control cells, it significantly enhanced etoposide sensitivity of TLP-knockdown cells, making TLP-knockdown cells as sensitive as control cells to etoposide (Fig. 4e). These results suggest that HR repair is more active in TLP-knockdown cells and that this makes TLP-knockdown cells more resistant to etoposide.

### DSB is efficiently repaired in transcriptionally active TLP-knockdown cells

**Figure 5:**
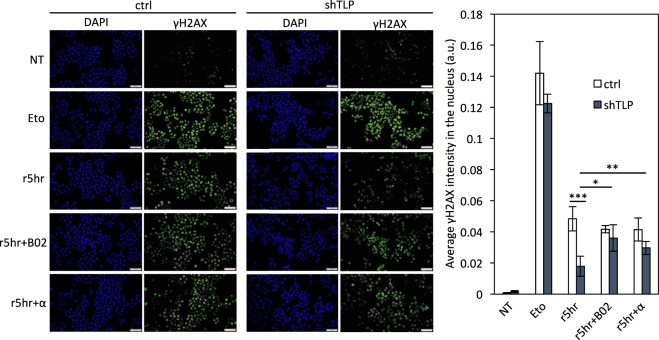
DSB is efficiently repaired in transcriptionally active TLP-knockdown cells. Control (ctrl) and TLP-knockdown (shTLP) HeLa cells were treated with 10 μM etoposide (Eto) for 30 min or left untreated (NT). Then, etoposide-treated cells were washed with PBS and incubated for 5 h in fresh medium (r5hr) with or without 20 μM B02 or 4 μg/ml α-amanitin before immunofluorescence staining of γH2AX. The nucleus was counterstained with DAPI. The fluorescent intensity of γH2AX was measured by using the CellProfiler software, and average intensity of γH2AX per nucleus area is shown in the right panel. *p < 0.05; **p < 0.01; ***p < 0.001; n.s, non-significant.

To obtain direct evidence that HR repair is accelerated in TLP-knockdown cells, we quantified γH2AX, a sensitive indicator of DSB^43^, after etoposide treatment of control and TLP-knockdown cells (Fig. 5). Gamma-H2AX foci were formed immediately after exposure to etoposide, and signal intensity gradually diminished after etoposide removal. Five hours after etoposide removal, signal intensity decreased by ~67% in control cells and by ~86% in TLP-knockdown cells from the respective peak levels (Fig. 5, Eto v.s. r5hr). The number of γH2AX foci per cell was also counted. Five hours after etoposide removal, ~80% of control cells still had more than 20 foci per cell, whereas ~55% of TLP-knockdown cells had more than 20 foci per cell (Supplementary Fig. S5a). These results suggest that etoposide-induced DSBs are repaired in TLP-knockdown cells more rapidly. While expression levels of nearly 300 genes were significantly up- or down-regulated by TLP knockdown (Supplementary Fig. S6a), none of the 54 genes involved in HR or NHEJ was significantly affected by TLP knockdown (Supplementary Fig. S6b), suggesting that rapid DSB repair in TLP-knockdown cells does not result from change in expression levels of DSB repair genes.

Finally, we examined the effects of B02 and the transcriptional inhibitors α-amanitin and DRB on the γH2AX level. In control cells, the γH2AX signal that remained 5 h after etoposide removal was not significantly affected by B02, α-amanitin, and DRB (Fig. 5 and Supplementary Fig. S5b). This implies that transcription is not required for DSB repair in control cells and that HR is not a major DNA repair pathway in etoposide-treated control cells. By contrast, B02, α-amanitin, and DRB all resulted in an increase of the γH2AX level in TLP-knockdown cells, suggesting that transcription is critical for DSB repair in TLP-knockdown cells, and that HR is a major DNA repair pathway in etoposide-treated TLP-knockdown cells.

## Discussion

**Figure 6:**
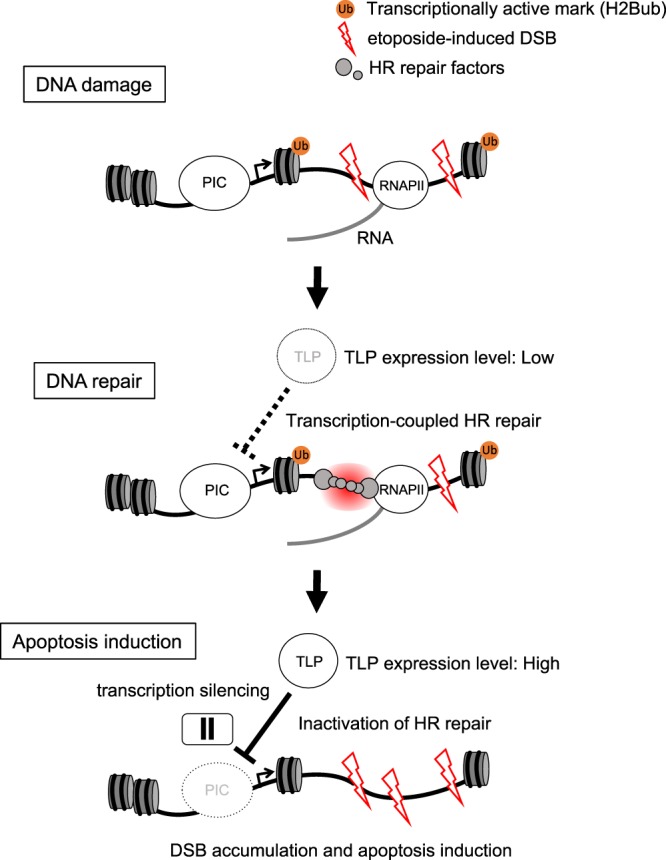
A model for DNA damage repair regulation by TLP-induced transcriptional repression. Prolonged exposure to etoposide results in an increase of TLP expression, leading to global inhibition of transcription initiation. HR repair is inactivated as a consequence of transcriptional repression, and this leads to accumulation of DSBs and apoptosis.

Here we revealed the mechanism by which TLP induces apoptosis through transcriptional repression after DNA damage (Fig. 6). TLP contributes to global transcriptional repression after etoposide exposure, thereby slowing down DSB repair and inducing apoptosis. Transcription is repressed by multiple mechanisms after DNA damage. For example, ATM-dependent ubiquitination of H2A causes transcriptional repression through chromatin remodeling^6,8^. Moreover, it is shown that the recruitment of Polycomb repressive complex 1 to DSB sites contributes to DNA damage-dependent transcriptional repression^9^. While most of the mechanisms described so far involve the inhibition of transcription elongation, the mechanism uncovered in this study involves the inhibition of transcription initiation, implying that DNA damage causes efficient transcriptional inhibition through multiple mechanisms. It is therefore not surprising that etoposide-induced transcriptional repression was only partially reversed by TLP knockdown (Fig. 3). While the TLP protein level is usually maintained at a low level, its expression is significantly induced by etoposide exposure by an as yet unknown mechanism (Fig. 1). On the other hand, our knockdown study showed that TLP represses the transcription of target genes before and after etoposide treatment (Fig. 3). It is therefore plausible that TLP serves as a transcriptional repressor not only in the late stage but also in the early stage of etoposide-induced cellular responses and regulates the processes. More specifically, we speculate that the basal level of TLP slows down DSB repair in the early stage of etoposide treatment and that, when induced by sustained DNA damage, TLP potentiates its function as a transcriptional repressor and accelerates apoptosis to cells that have sustained DNA damage (Fig. 6).

Despite attention, the interplay between transcription and DSB repair is not fully understood. It is considered that transcriptional repression is beneficial for efficient DSB repair, as actively elongating RNAPII could be an impediment to DSB repair machinery^44^. Concordantly, it has been reported that inhibition of transcription elongation around damaged sites results in efficient DSB repair^8^. Other studies have shown, however, that sustained transcription is critical for the recruitment of DNA repair factors to damaged sites, and that nascent RNA around damaged sites facilitates HR repair^2,4^. Taking these findings into account, it is plausible that, while DSB results in the inhibition of transcription elongation, open chromatin structure allows faster access of DNA repair factors to damaged sites, and that nascent RNA associated with arrested RNAPII is used as a template for HR repair, all leading to efficient DNA repair. Given this assumption, TLP-mediated transcriptional repression seems to inhibit DSB repair through global changes to histone modifications and/or through a reduction of chromatin-associated nascent RNA. In support, the total H2Bub level and the level of ongoing transcription after etoposide exposure were higher in TLP-knockdown cells than in control cells (Figs 3 and 4). TLP-knockdown cells showed resistance to etoposide, doxorubicin, and γ irradiation, but not to MMS and MG132, suggesting that there is some specificity in the action of TLP. Unlike MMS, which is mainly repaired by the base excision repair pathway, DSBs induced by etoposide, doxorubicin, and γ irradiation are preferentially repaired by the HR pathway. It is therefore likely that TLP is particularly important in the regulation of transcription-coupled HR repair.

What is the physiological significance of TLP-mediated inhibition of DSB repair? Its possible role includes cancer prevention through elimination of DNA-damaged cells. According to this scenario, TLP expression is increased by sustained DNA damage and triggers the shift from the DNA repair mode to apoptosis in severely damaged cells. A prediction from this study is that cells expressing TLP at a low level are resistant to DSB-induced apoptosis and therefore tumorigenic. Intriguingly, previous microarray analyses of clinical samples indicate that, in testis, in which TLP is normally expressed at a high level, TLP expression is significantly reduced in cancerous tissues such as yolk sac tumor, teratoma, and embryonal carcinoma^45^. Here we demonstrated that a reduced expression of TLP confers resistance to Topo II poisons. Since etoposide is used for the treatment of various types of cancer, including testicular cancer, our findings may have clinical relevance. TLP might be useful as a prognostic marker or as a predictor of chemotherapy efficacy.

## Methods

### Cell culture and drug treatment

Control HeLa cells and TLP-knockdown HeLa cells^31^ were maintained in Dulbecco’s modified Eagle’s medium containing glucose and 10% fetal calf serum at 37 °C. shTLP-TetOn cells, which harbor the Tet-On expression system for FLAG/polyhistidine-tagged TLP (FH-TLP), were maintained in medium containing glucose, 10% fetal calf serum, and puromycin. Etoposide, doxorubicin, MG132, and B02 were dissolved in dimethyl sulfoxide (DMSO) and used at indicated concentrations. Cell viability was assayed using Cell Count Reagent SF (Nacalai Tesque).

### Quantitative reverse-transcription PCR (qRT-PCR)

Total cellular RNAs were prepared from HeLa cells using RNeasy Mini Kit (Qiagen) as described by the manufacturer, quickly frozen, and stored at −80 °C until use. Quantification of purified RNA was performed using NanoDrop One (Thermo Fisher Scientific). Reverse transcription was performed using 1 µg of total RNA, SuperScript III Reverse Transcriptase (Thermo Fisher Scientific), and Oligo(dT)_20_ (Thermo Fisher Scientific) as described by the manufacturer, without prior DNase treatment. It was confirmed that little or no signal was detected by qPCR without reverse transcriptase. qPCR analysis was performed by using StepOnePlus Real-Time PCR System (Thermo Fisher Scientific), KAPA SYBR FAST qPCR Master Mix (KAPA Biosystems), and 1 µl of RT products as described by the manufacturer. Sequences and information of the primers used for qPCR are listed in Supplementary Table S1. Linear amplification of PCR products was confirmed by serial dilution of template DNA, and quantification was made within the linear range of amplification. Most of the primer sets used in this study have nearly 2.0-per-cyce amplification efficiency with negligible y-intercept. Most of the coefficients of determination derived from standard curves were greater than 0.98. Specific amplification was confirmed by melting curve analysis and agarose gel electrophoresis. qPCR data were analyzed by StepOne Software v2.2.2, and relative quantification was made by the ∆Ct method.

### Western blotting

Proteins were separated by SDS-PAGE, transferred to an Immobilon-P PVDF membrane (Millipore), and detected by ECL Western Blotting Detection Reagents (GE Healthcare). The following primary antibodies were used: anti-p53 (Santa Cruz, sc-6243), anti-β-actin (Santa Cruz, sc-47778), anti-Myc (Santa Cruz, sc-789), anti-Caspase 3 (Cell Signaling Technologies, 9662), anti-Bcl-xL (Cell Signaling Technologies, 2764), anti-H2B-K123 monoubiquitination (Cell Signaling Technologies, 5546), anti-H2B (Abcam, ab1790), anti-TLP^18^, anti-TFIIAγ^23^. Anti-RNAPII CTD, anti-RNAPII CTD-S2p, and anti-RNAPII CTD-S5p^46^ were kind gifts from Hiroshi Kimura (Tokyo Institute of Technology).

### Immunofluorescence microscopy

One day before immunofluorescence experiments, cells were seeded on cover slips. Cells were fixed with 4% paraformaldehyde for 5 min, permeabilized with 0.5% Triton X-100 for 10 min, and blocked with Blocking One (Nacalai Tesque) for 1 h at room temperature. Cells were then incubated with primary and Alexa Fluor 488-conjugated secondary antibodies for 1 h each. After counterstained with DAPI, cover slips were mounted on glass slides. Fluorescent images were obtained using a fluorescence microscope CKX53 (Olympus) and analyzed using the CellProfiler software.

### Chromatin immunoprecipitation (ChIP)

Cross-linking was performed with 1% formaldehyde for 15 min. Cross-linked cells were washed with ice-cold PBS containing 0.125 M glycine and then with hypotonic buffer (10 mM Hepes-KOH (pH7.8), 10 mM KCl, 0.1 mM EDTA, and 0.1% NP-40) containing protease inhibitors and pelleted by centrifugation. The nuclear pellet was resuspended with MNase Reaction Buffer (20 mM Tris-HCl (pH 8.0), 5 mM NaCl, 2.5 mM CaCl_2_, and 0.05 U/µl MNase) and incubated at 37 °C for 20 min for chromatin fragmentation. After centrifugation, the pellet was resuspended again with ChIP Lysis Buffer (1% SDS, 10 mM EDTA, and 50 mM Tris–HCl (pH 8.0)) and subjected to mild sonication. The lysate was diluted five-fold using ChIP Dilution Buffer (1% Triton X-100, 1.2 mM EDTA, 167 mM NaCl, and 16.7 mM Tris–HCl (pH 8.0)) and precleared for 1 h at 4 °C with pre-blocked Protein G Sepharose beads (GE Healthcare). Precleared lysates containing 25 μg of DNA per reaction were incubated with specific antibodies overnight at 4 °C with rotation. Twenty microliters of Protein G Sepharose beads were added to each sample and further incubated for 3 h at 4 °C with rotation. Beads were washed once with Low Salt Buffer (0.1% SDS, 1% Triton X-100, 2 mM EDTA, 150 mM NaCl, and 20 mM Tris–HCl (pH 8.0)), once with High Salt Buffer (0.1% SDS, 1% Triton X-100, 2 mM EDTA, 500 mM NaCl, and 20 mM Tris–HCl (pH 8.0)), once with LiCl Buffer (0.25 M LiCl, 1% NP-40, 1% sodium deoxycholate, 1 mM EDTA, and 10 mM Tris–HCl (pH 8.0)), and twice with TE Buffer (1 mM EDTA and 10 mM Tris–HCl (pH 8.0)). DNA-protein complexes were eluted with 250 μl of Elution Buffer (1% SDS, 0.1 M NaHCO_3_, and 10 mM DTT). Cross-links were reversed by heating at 65 °C in the presence of 0.2 M NaCl at least for 8 h, followed by RNase A treatment at 37 °C for 0.5 h and Proteinase K treatment at 55 °C for 2 h. DNA was purified by phenol-chloroform extraction and ethanol precipitation and dissolved in water. Quantification of purified DNA was performed using NanoDrop One (Thermo Fisher Scientific). ChIP signals were detected by qPCR using StepOnePlus Real-Time PCR System, KAPA SYBR FAST qPCR Master Mix, and the primer sets listed in Supplementary Table S1. Linear amplification of PCR products was confirmed by serial dilution of template DNA, and quantification was made within the linear range of amplification. Specific amplification was confirmed by melting curve analysis and agarose gel electrophoresis. qPCR data were analyzed by StepOne Software v2.2.2, and relative quantification was made by using the ∆Ct method.

### 4-thiouridine (4sU)-RNA labeling

RNA labeling with 4sU and its purification was performed essentially as described previously^47^. Briefly, nascent RNAs were labelled by incubating cells with 500 μM 4sU (Sigma) for 45 min, immediately extracted with Sepasol-RNA I Super G (Nacalai Tesque), quickly frozen, and stored at −80 °C until use. Seventy micrograms of purified RNA was incubated with EZ-Link Biotin-HPDP (Thermo Fisher Scientific) in Biotinylation Buffer (10 mM Tris (pH 7.4) and 1 mM EDTA) for 1.5 h at room temperature. Following phenol-chloroform extraction and ethanol precipitation, biotinylated RNAs were isolated by µMACS Streptavidin beads (Miltenyi Biotec), eluted with 100 mM DTT, and further purified using RNeasy MinElute Cleanup Kit (Qiagen). The quantity of recovered RNA was measured using a Quantus fluorometer (Promega). qRT-PCR analysis was performed by using StepOnePlus Real-Time PCR System (Thermo Fisher Scientific), One Step TB Green Prime Script RT-PCR (TaKaRa), and the primer sets listed in Supplementary Table S1.

### mRNA-sequencing (mRNA-seq) and data analysis

Ribosomal RNA was removed from samples using RiboMinus Eukaryote Kit (Thermo Fisher Scientific), and libraries for mRNA-seq were prepared using Ion Total RNA-seq Kit v2 (Thermo Fisher Scientific). The sizes of fragmented RNA and library cDNA were determined using the MultiNA microchip electrophoresis system (Shimadzu), and the quantity of library cDNA was determined by qPCR using KAPA Library Quantification Kit (KAPA Biosystems). mRNA-seq was performed using the Ion Proton sequencer and Ion PI Chip v3 (Thermo Fisher Scientific). Mapping of sequenced reads to the human genome reference hg19 and calculation of fragments per kilobase of transcript per million mapped reads (FPKM) were performed using the software CLC Genomic Workbench (Qiagen). All data were deposited in NCBI’s Gene Expression Omnibus and are publicly accessible through GEO accession number GSE120734. Fold change of gene expression was calculated using the following equation. DAVID 6.8 was used for gene ontology (GO) analysis.

$${\rm{Fold}}\,{\rm{change}}=({{\rm{FPKM}}}_{{\rm{etoposide}}}+1)/({{\rm{FPKM}}}_{{\rm{control}}}+1)$$

### Flow cytometry

Trypsinized and PBS-washed cells were fixed with ethanol, treated with 0.5 mg/ml RNase A, and stained with 50 μg/mL propidium iodide as described previously^32^. Cell cycle was analyzed by using a FACSCalibur flow cytometer and the ModFit LT software (BD Biosciences).

### ^60^Co γ irradiation

Exponentially growing cells were irradiated with cumulative doses of 25, 50, and 100 Gy using a ^60^Co source at a dose rate of 2 kGy/h. After irradiation, cells were and maintained at 37 °C for 72 h, and then cell number was determined by using a Countess automated cell counter (Life Technologies).

### Statistical analysis

Data obtained in this study are shown as means ± standard deviation (S.D.) from at least three independent experiments. Statistical significance was determined by Student’s t-test or Bonferroni’s method using the R Console version 3.0.3. p < 0.05 was regarded as statistically significant. Statistical significance is indicated in the figures with asterisks as follows: *p < 0.05; **p < 0.01; ***p < 0.001.

## Supplementary information

Supplementary Information

## Data Availability

The datasets generated during and/or analyzed during the current study are available in the NCBI Gene Expression Omnibus, https://www.ncbi.nlm.nih.gov/geo/query/acc.cgi?acc=GSE120734.
