# Supplementary material for: TLP-mediated global transcriptional repression after double-strand DNA breaks slows down DNA repair and induces apoptosis
Source: Sci Rep. 2019 Mar 19;9:4868. doi: 10.1038/s41598-019-41057-9 (PMC6425004; doi:10.1038/s41598-019-41057-9)
Supplement: Supplementary file 1 — Supplementary Information [file 41598_2019_41057_MOESM1_ESM.pdf]

TLP-mediated global transcriptional repression after double-strand DNA breaks slows down DNA repair and induces apoptosis

Hidefumi Suzuki[1], Mayumi Okamoto-Katsuyama[1], Tetsufumi Suwa[1], Ryo Maeda[2], Taka-aki Tamura[2], and Yuki Yamaguchi[1*]

[1] School of Life Science and Technology, Tokyo Institute of Technology, 4259 Nagatsuta, Yokohama 226-8501, Japan.
[2] Graduate School of Science, Chiba University, 1-33 Yayoicho, Chiba 263-8522, Japan.

* To whom correspondence should be addressed.
Tel: +81-45-924-5798. Fax: +81-45-924-5834
Email: yyamaguc@bio.titech.ac.jp

Figure-S1 (Suzuki et al.)

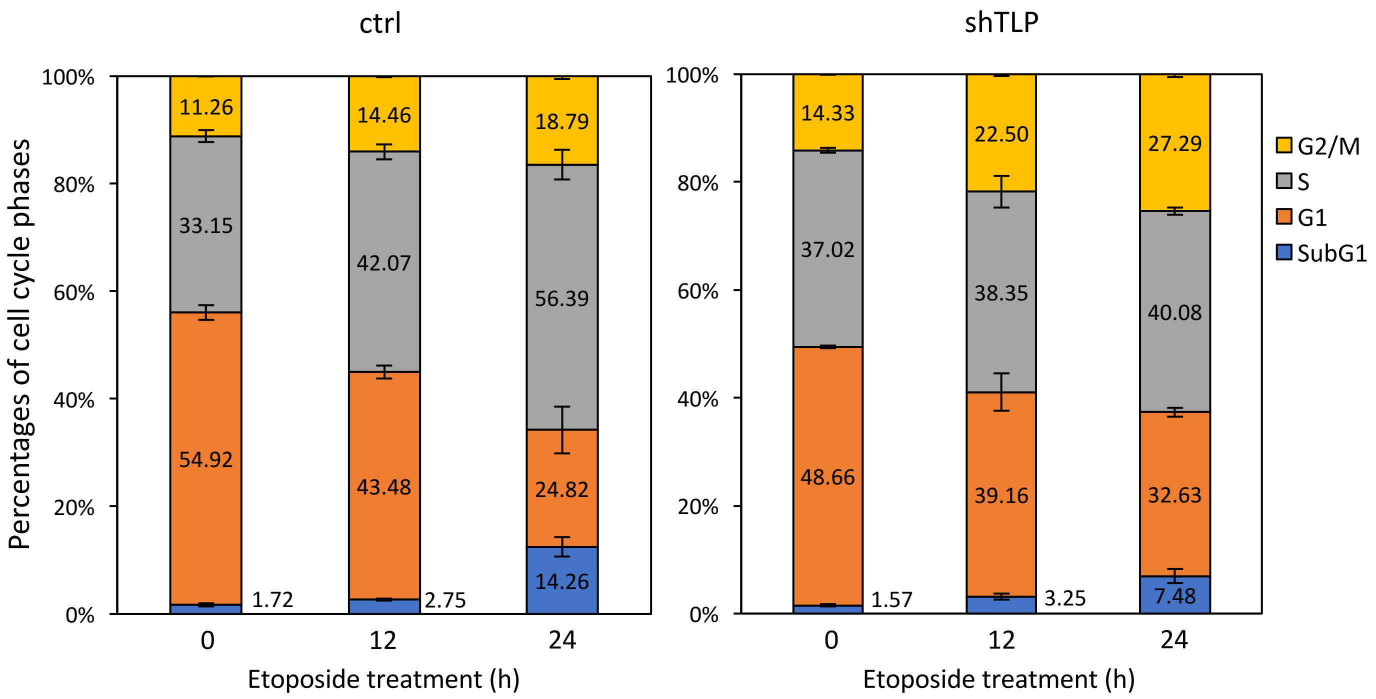

Supplementary Figure S1. Cell cycle analysis of etoposide-treated cells.
Control (ctrl) and TLP-knockdown (shTLP) HeLa cells were treated with 50 μM etoposide for indicated periods, and proportions of cell cycle phases under each condition were determined by flow cytometry.

Figure-S2 (Suzuki et al.)

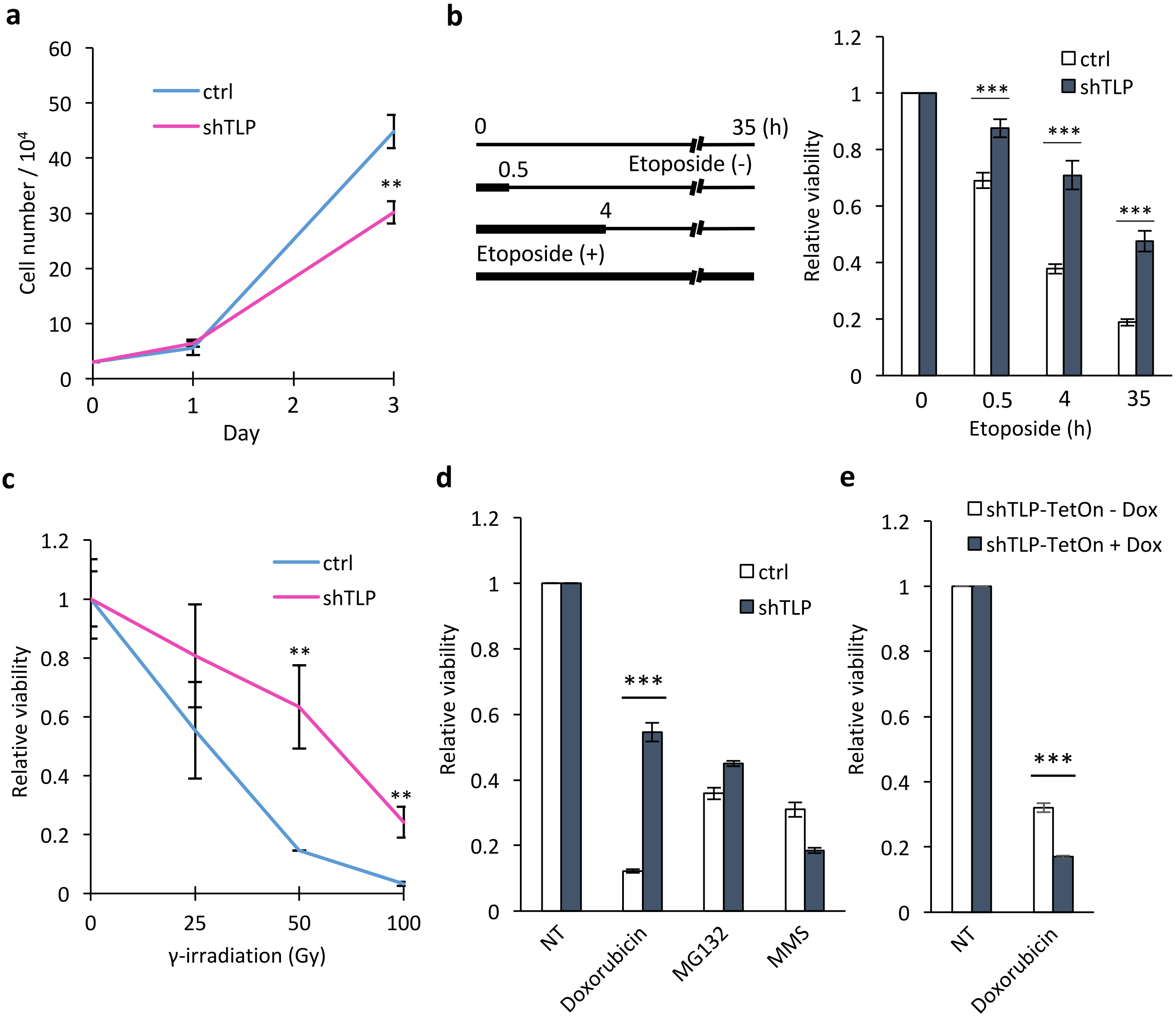

Supplementary Figure S2. DNA-damage sensitivity of TLP-knockdown cells.
(a) Cell proliferation rates of control (ctrl) and TLP-knockdown (shTLP) HeLa cells were compared. The number of cells were counted at indicated times. (b) Cell viability analysis after short-time etoposide treatment. Control (ctrl) and TLP-knockdown (shTLP) HeLa cells were treated with 50 μM etoposide for indicated periods and then recovered in its absence. SF assay was performed 35 h after etoposide addition. The time courses of etoposide treatment are shown to the left. (**c**) Cell viability analysis after γ-irradiation. Control (ctrl) and TLP-knockdown (shTLP) HeLa cells were irradiated with indicated doses of γ-rays. Cell numbers were determined 72 h after γ-irradiation. (**d**) Control (ctrl) and TLP-knockdown (shTLP) HeLa cells were treated with 2 μM doxorubicin, 0.3 mM methyl methanesulfonate (MMS), or 0.5 μM of MG-132 for 36 h or left untreated (NT), and cell viabilitiy was determined by SF assay. (**e**) The effect of TLP overexpression on doxorubicin sensitivity. TLP overexpression was induced by adding 100 ng/ml doxycycline (Dox) to shTLP-TetOn cells. Control and TLP-overexpressing cells were treated with 2 μM doxorubicin for 24 h or left untreated, and cell viability was determined by SF assay. Data were normalized to the level of nontreated cells and represent the average and S.D. of three independent experiments. **, p < 0.01; ***, *p* < 0.001.

Figure-S3 (Suzuki et al.)

a

Eto-Down genes in control cells	P-Value
nucleosome assembly	9.62E-18
cell-cell adhesion	8.73E-17
gene silencing by RNA	2.94E-14
chromatin silencing at rDNA	6.52E-13
positive regulation of gene expression, epigenetic	1.73E-12

Eto-Down genes in TLP-knockdown cells	P-Value
nucleosome assembly	2.59E-17
cell-cell adhesion	4.37E-16
gene silencing by RNA	9.63E-15
positive regulation of gene expression, epigenetic	3.08E-14
chromatin silencing at rDNA	6.64E-14

Eto-Up genes in control cells	P-Value
type I interferon signaling pathway	9.24E-15
defense response to virus	1.01E-13
negative regulation of viral genome replication	6.33E-09
innate immune response	2.44E-07
interferon-gamma-mediated signaling pathway	1.61E-06

Eto-Up genes in TLP-knockdown cells	P-Value
type I interferon signaling pathway	1.57E-13
defense response to virus	6.71E-10
interferon-gamma-mediated signaling pathway	7.61E-09
negative regulation of viral genome replication	8.28E-08
inflammatory response	1.15E-05

b

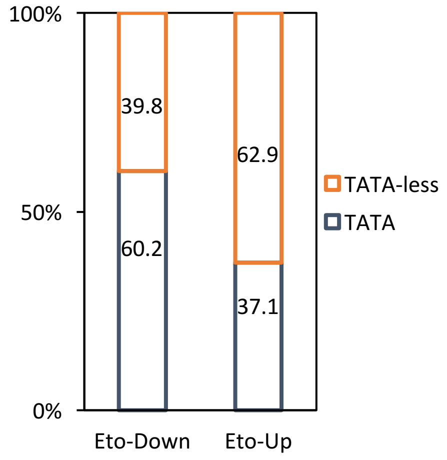

Supplementary Figure S3. mRNA-seq data analysis.
(**a**) GO analysis of top 1,000 etoposide-downregulated and -upregulated genes in control and TLP-knockdown cells. (**b**) Top 1,000 genes downregulated (Eto-Down) or upregulated (Eto-Up) by etoposide in control cells were categorized into TATA-containing or TATA-less genes by DAVID 6.8, and the fractions of each gene group are shown. Genes were ranked by fold changes of FPKM.

Figure-S4 (Suzuki et al.)

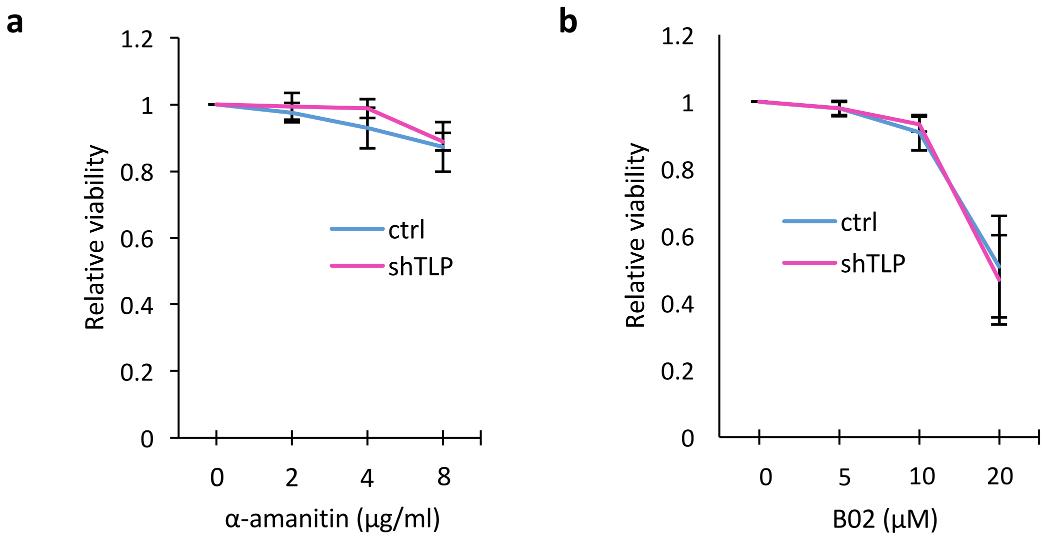

Supplementary Figure S4. Effects of α-amanitin and B02 on cell viability.
(**a**) Control (Ctrl) and TLP-knockdown (shTLP) HeLa cells were treated with indicated concentrations of α-amanitin for 24 h, and cell viability was determined by SF assay. (**b**) Control and TLP-knockdown HeLa cells were treated with indicated concentrations of B02 for 36 h, and cell viability was determined by SF assay. Data were normalized to the level of nontreated cells and represent the average and S.D. of three independent experiments.

Figure-S5 (Suzuki et al.)

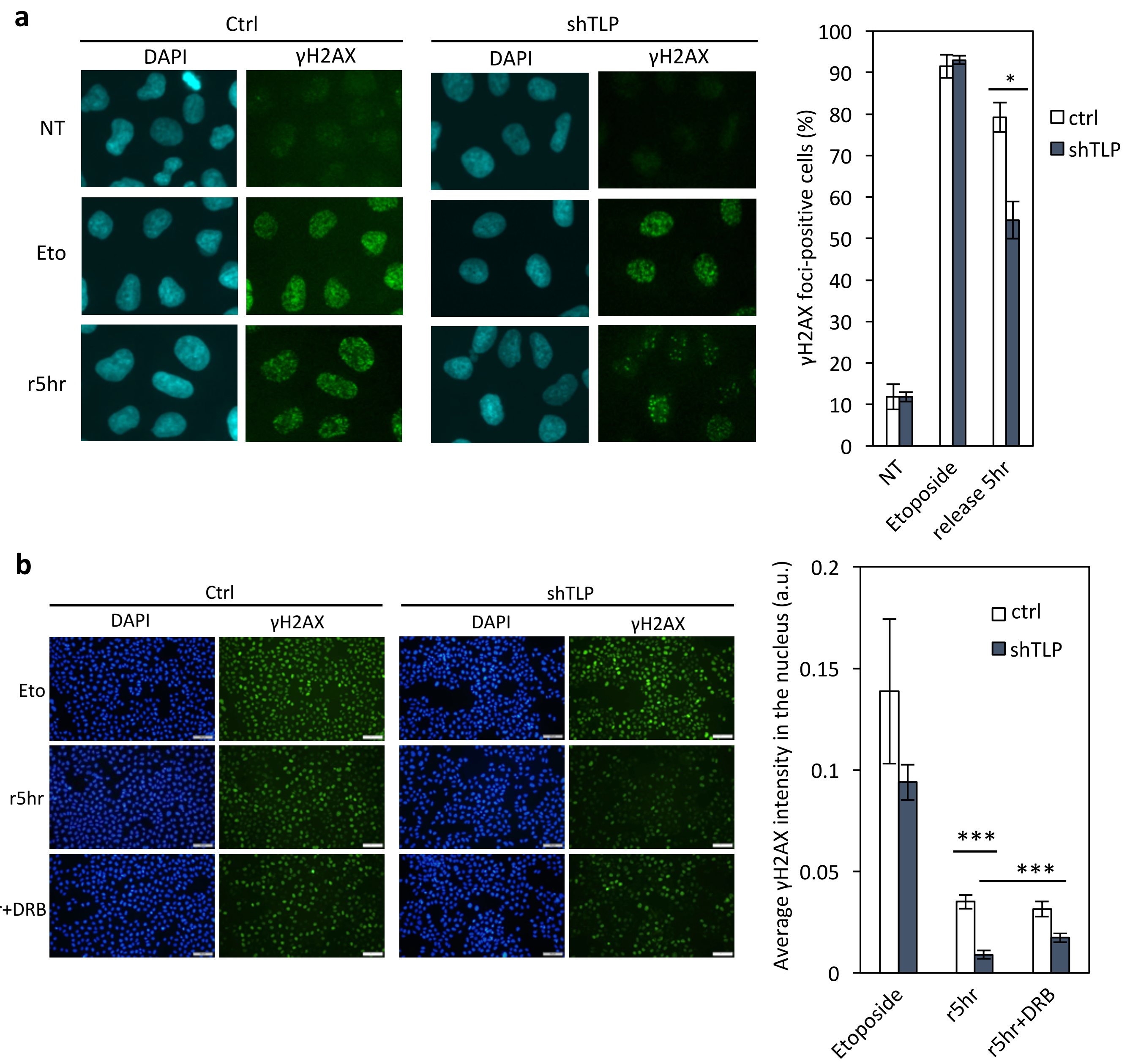

Supplementary Figure S5. Effects of DRB-mediated transcription inhibition on DSB repair.
(**a**) Control (Ctrl) and TLP-knockdown (shTLP) cells were treated with 10 µM etoposide (Eto) for 30 min or left untreated (NT). Then, etoposide-treated cells were washed with PBS and incubated for 5 h in fresh medium (r5hr) before immunofluorescence staining of γH2AX. The nucleus was counterstained with DAPI. The number of cells with more than 20 γH2AX foci was counted, and the percentages of γH2AX foci-positive cells are shown in the right panel. The data represent the average and S.D. of three independent experiments. (**b**) Control and TLP-knockdown cells were treated with 10 µM etoposide for 30 min and then recovered for 5 h in the absence (r5hr) or presence (r5hr+DRB) of 50 µM DRB before immunofluorescence staining. The fluorescent intensity of γH2AX was measured by Cell Profiler, and relative average intensity of γH2AX per nucleus area is shown in the right panel (n=3). *, $p<0.05$; ***, $p<0.001$.

Figure-S6 (Suzuki et al.)

a

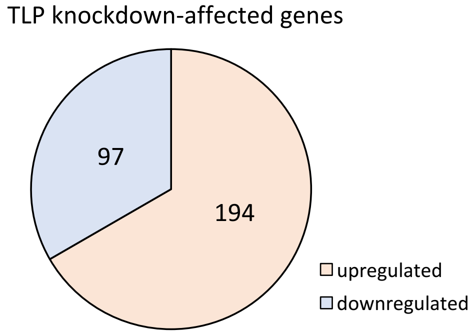

TLP knockdown-affected genes	P-Value
translation	2.86E-07
cholesterol biosynthetic process	1.61E-05
cell-cell adhesion	2.62E-05
negative regulation of protein serine/threonine kinase activity	2.43E-04
rRNA processing	4.37E-04
DNA replication	6.97E-04
cell redox homeostasis	8.58E-04
DNA replication initiation	1.18E-03
response to hypoxia	1.93E-03
protein targeting to mitochondrion	1.97E-03

b

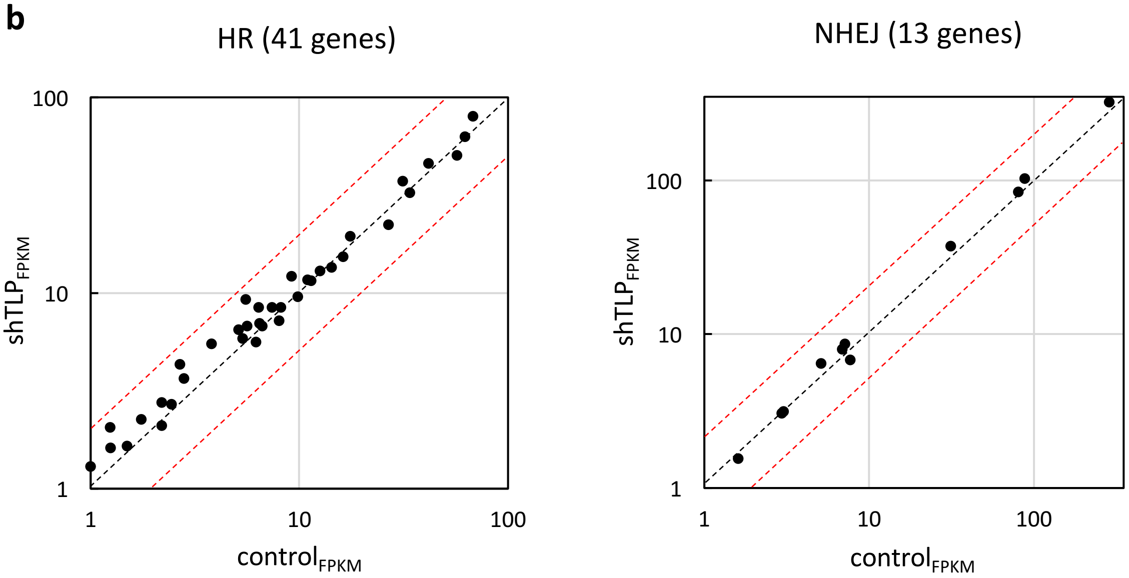

Supplementary Figure S6. Expression of DSB repair genes is not significantly affected by TLP knockdown.

(**a**) Gene expression levels were compared between control and TLP-knockdown cells under DMSO-treated condition. Genes with $p < 0.05$ and $\log_2|\text{fold change}| \geq 1.0$ were regarded as TLP-knockdown-affected genes. The numbers of upregulated and downregulated genes were shown in pie chart (left). Also, GO analysis was performed for all 291 genes of TLP-knockdown-affected genes.

(**b**) Expression levels of the genes involved in HR (41 genes) and NHEJ (13 genes) were compared between control and TLP-knockdown cells under DMSO-treated condition using the mRNA-seq data. None of the 54 genes involved in HR or NHEJ is significantly affected by etoposide, as judged by the criteria used for the determination of etoposide-affected genes ($p < 0.05$ and $\log_2|\text{fold change}| \geq 1.0$)

Figure-S7 (Suzuki et al.)

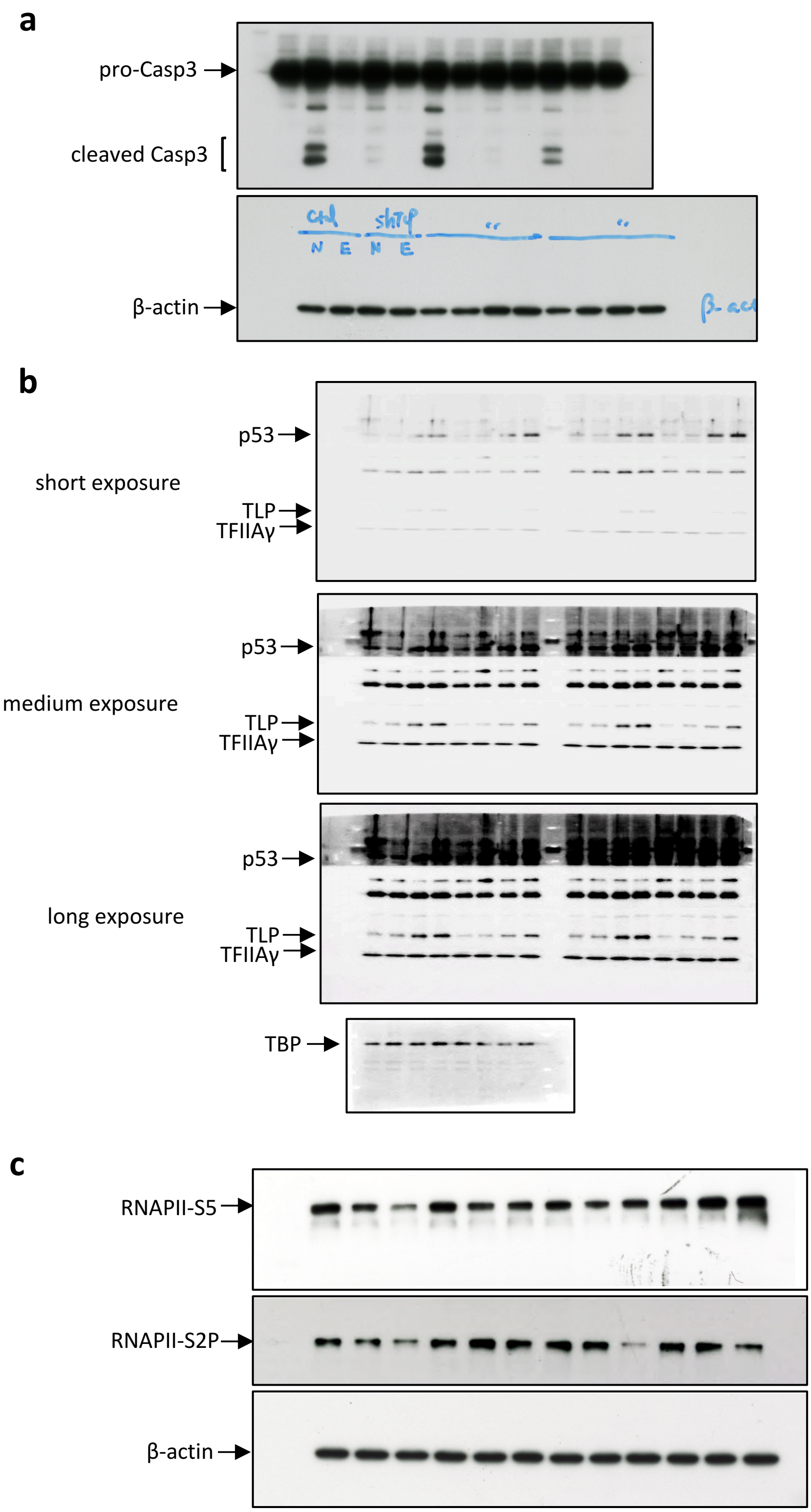

Supplementary Figure S7. Full-length Western blot images used for cropped images presented in Fig. 1b (a), Fig. 1d (b) and Fig. 1e (c).

Figure-S8 (Suzuki et al.)

a

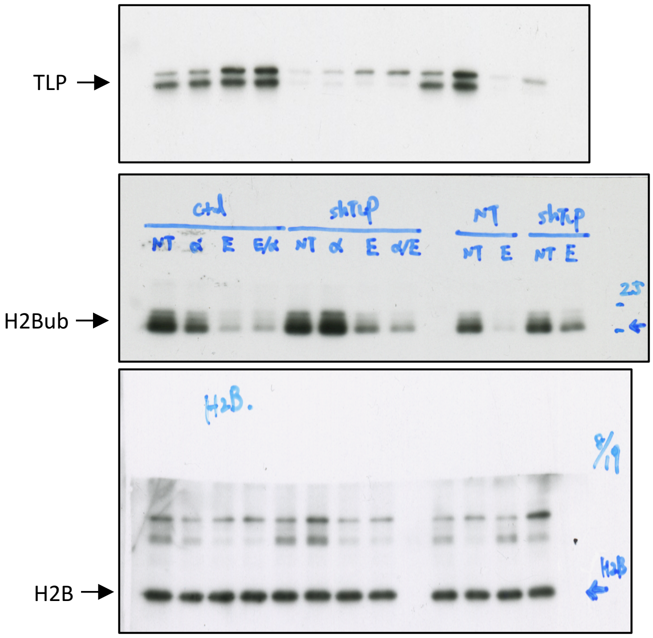

b

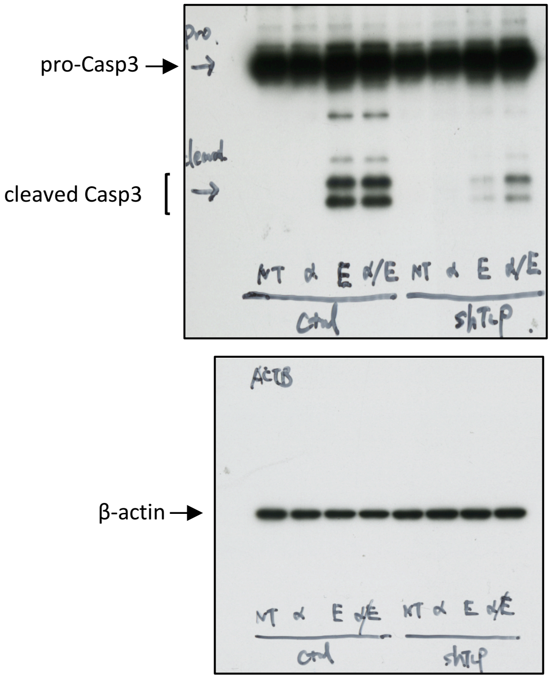

Supplementary Figure S8. Full-length Western blot images used for cropped images presented in Fig. 4a, Fig. 4b (a), and Fig. 4d (b).

Supplementary Table S1

Primer sequences and information

Experiment	Region	Forward primer (5'-3') (location)	Reverse primer (5'-3') (location)	Accession number	Amplicon size (bp)	PCR efficiency (%)	R2 of standard curve	Targeted transcript variant
qRT-PCR	ZDHHC24	GGGCCAGCACTCCTATGACC (exon3)	CTGTGGTCTGGAAGGTGATCC (exon3)	Gene ID: 254359	135	95	0.987	-
	GAPDH	GTCAAGGCTGAGAACGGGAA (exon4)	AAATGAGCCCCAGCCTTCTC (exon5)	Gene ID: 2597	159	95	0.993	1, 2, 3, 4, 6, 7
	MB21D2	GTTGCTCTCTGGAGGTGTCC (exon2)	CATGTCGAGTGTCACAGGCT (exon2)	Gene ID: 151963	174	99	0.982	-
	EPAS1	AGCTGACAAGGAGAAGAAAAGGA (exon1/2)	TGGGCCAGCTCATAGAACAC (exon2)	Gene ID: 2034	112	97	0.996	-
	SUOX	CCGACGCTCTGAGATGACTC (exon6)	AGCCTGGGCTAACACATCAC (exon6)	Gene ID: 6821	115	94	0.982	1, 2, 3
	TLP	GGAAGATTGCTTTGGAAGGAGC (exon2)	CCTGAGGACCAAATTGTAGCTG (exon3)	Gene ID: 9519	106	90	0.961	1, 2
	HPDL	CACTTTTGCGCTGGTTCCAC (exon1)	ACCCTGCTGTCATTTCGAGG (exon1)	Gene ID: 84842	96	100	0.953	-
	rDNA	AGTCGGGTTGCTTGGGAATGC	CCCTTACGGTACTTGTTGACT	NT_167214.1	97	106	0.996	-
ChIP	ACTB	TCCTCAATCTCGCTCTCGCT	GCCGCTGGGTTTTATAGGGC	NC_000007.14	182	99	0.996	-
	GAPDH	CTCAAGACCTTGGGCTGGG	TCGAACAGGAGGAGCAGAGA	NC_000012.12	132	114	0.991	-
	rDNA	AGTCGGGTTGCTTGGGAATGC	CCCTTACGGTACTTGTTGACT	NT_167214.1	97	106	0.996	-
	EPAS1	CGCCCAGACGACCTCATAAA	TCCCGGCCAATGGGGA	NC_000002.12	124	103	0.998	-
	Intergenic region	CCCTCTAGTGCTTCATTTGACCT	TTCTTAAGGGCAGTCACAGTCTT	NC_000001	142	115	0.986	-
